# Designs of NKG2D-based immunotherapeutics for cancer

**DOI:** 10.3389/fimmu.2025.1557644

**Published:** 2025-06-19

**Authors:** Jianfeng Han, Youwei Wang, Godfrey Chi-Fung Chan, Wing Keung Chan

**Affiliations:** ^1^ Division of Hematology, Department of Internal Medicine, College of Medicine, The Ohio State University, Columbus, OH, United States; ^2^ Institute of Medical Engineering & Translational Medicine, Tianjin University, Tianjin, China; ^3^ Department of Pediatric and Adolescent Medicine, Li Ka Shing Faculty of Medicine, The University of Hong Kong, Hong Kong, Hong Kong SAR, China; ^4^ Centre of Paediatric Hematology and Oncology, Hong Kong Sanatorium and Hospital, Hong Kong, Hong Kong SAR, China

**Keywords:** NKG2D, immunotherapy, CAR-T therapy, NK cell therapy, cancer

## Abstract

Natural killer group 2 D (NKG2D) receptor, one of the activation receptors on NK cells, has gained increasing attention in recent years because its ligands are widely expressed in most cancers. Naturally, NKG2D reacts to 8 different stress-induced ligands, MICA/B, and ULBP1-6. Despite being genomically conserved between human and mouse, NKG2D transcripts have splice variants that can differentiate the two. hNKG2D or mNKG2D (both long and short transcripts) interacts with DAP10 only in human but DAP10/12 in mouse, switching on different effector functions such as IFN-γ production and cytotoxicity. Full-length, extracellular or cytoplasmic domains have been used to construct chimeric antigen receptors (CAR) or implement into the antibody structures including bispecific antibodies. Interestingly, most of the NKG2D CARs, either on T cells or NK cells are investigated in preclinical models of solid tumors. In this article, we reviewed the majority of published NKG2D-based CAR and antibody designs, comparing their respective advantages and disadvantages. We also elaborated how these CARs and antibodies were tested in preclinical cancer models and clinical trials in this review article.

## 1 Introduction

**Figure 1:**
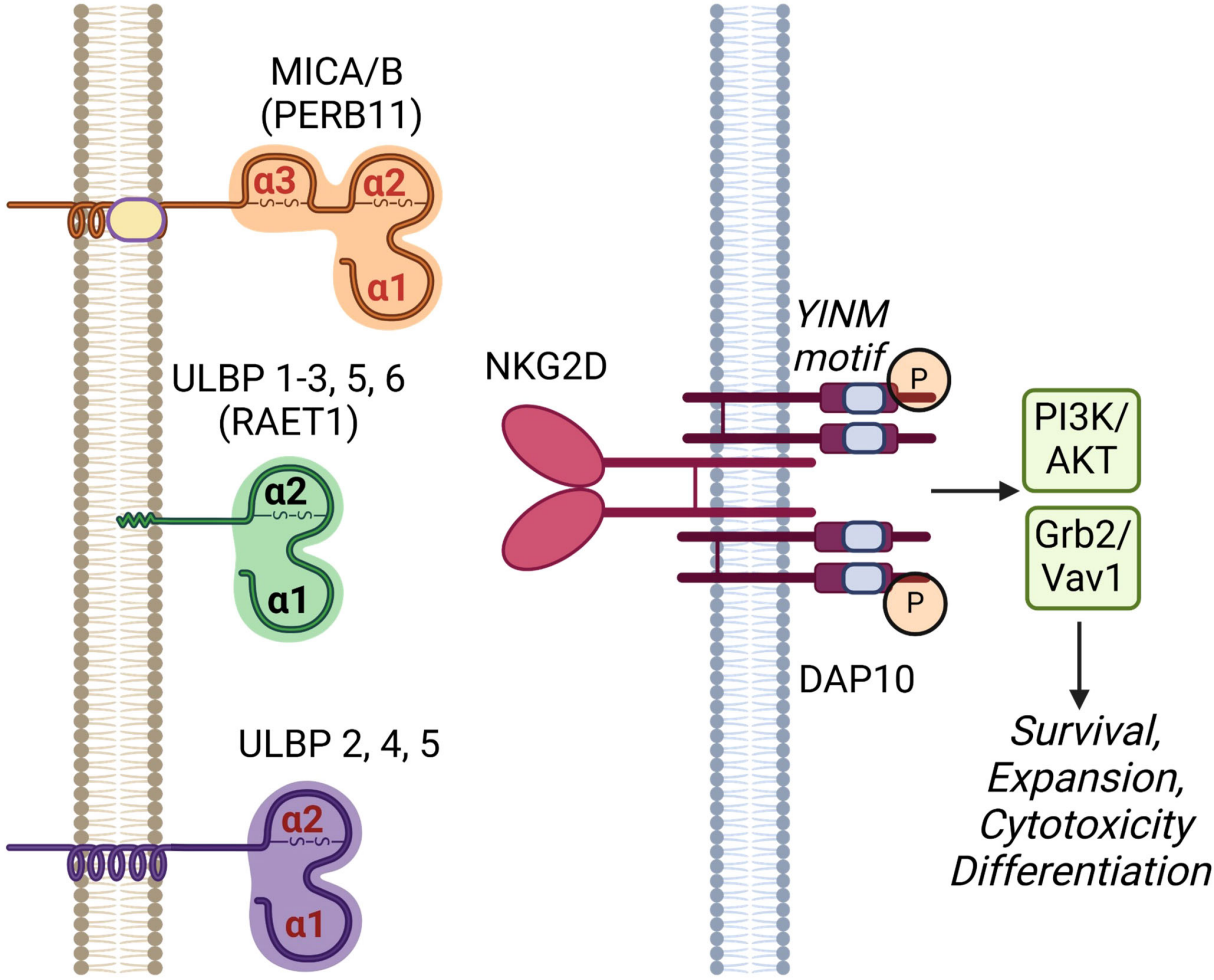
Human NKG2D receptor and its cognate ligands. NKG2D receptor is a C-type lectin-like molecule expressed primarily on NK cells and subsets of T cells. It has a disulfide-linked homodimer that is associated with four DNAX-activating protein 10 (DAP10) forming a hexameric complex. Upon phosphorylation, the YINM motifs of DAP10 recruit and activate phosphatidyl-inositol 3-kinase (PI3K)/Akt and Grb2/Vav1 molecules leading to cell survival, expansion, cytotoxicity and differentiation. NKG2D ligands include proteins in MHC class-I polypeptide-related sequence (MIC) and UL16-binding protein (ULBP) families. MICA and B (other names PERB11.1/11.2) have three MHC-class I related α1, α2 and α3 domains anchored on cell surface by a transmembrane domain. ULBP 1-6 (other name RAET1I/H/N/E/G/L) consists of only α1 and α2 domains and are attached to the cell membrane via either a glycosylphosphatidylinositol (GPI)-anchor (ULBP1-3, 5 and 6) or transmembrane domains (ULBP 2, 4, and 5). Created in BioRender. Chan, W. (2025) https://BioRender.com/n95p848.

NKG2D (gene name: *klrk1*) is one of the key activation receptors on NK cells and CD8+ T cells targeting cancer cells and infections (1–4). It is expressed on nearly all human NK and CD8+ T cells and the expression level can be upregulated by IL-2, 7, 12, 15 and negatively regulated by TGF-β, IFN-β1 and IL-21 (5, 6). Studies show NKG2D activation signals are sufficient to activate NK cell function in cytokine-primed NK cells and synergizes with other NK cell activation receptors including NKp46 and co-receptor 2B4 (5, 7). NKG2D recognizes eight stress-induced NKG2D ligands (NKG2DLs) including the MHC class I chain-related proteins A and B (MICA, MICB), and the structurally diverse UL16-binding proteins 1 to 6 (ULBP1-6) as shown in **Figure 1** (5, 8). A comparison of human and mouse NKG2D receptors reveals several distinctive features. Mice express two alternatively spliced isoforms of NKG2D: NKG2D-L (long) and NKG2D-S (short) (5). NKG2D-L is expressed on the surface of both resting and activated mouse NK cells and CD8 T cells as a disulfide-bonded homodimer that can interact with DAP10 homodimers. NKG2D-S is expressed on activated mouse NK cells, which can associate with homodimers of either DNAX activating protein (DAP) 10 or DAP12. DAP10 contains a tyrosine-based signaling motif, YINM, which is capable of recruiting a p85 PI3 kinase and Vav-1 signaling complex whereas DAP12 contains a canonical immunoreceptor tyrosine-based activation motif (ITAM), which can recruit Syk and ZAP70 tyrosine kinases (9, 10). In humans, NKG2D-L is the only one expressed on NK, CD8+, γΔ T and CD4+ iNKT cells (11). Upon engagement, the dimerized NKG2D leads to the phosphorylation of YINM motif of DAP10. Phosphorylated DAP10 will then activate both PI3K/AKT and Grb2/Vav1 axis resulting in the upregulations of survival signals, expansion, cytotoxicity and T cell differentiation (9).

The regulation of NKG2D ligands is tightly controlled and typically absent on healthy tissues, but can be upregulated due to DNA damage, infection, and cellular transformation under stress. In hematological malignancies and solid tumors, MICA or MICB is expressed in 100% of colorectal tumors, 97% of breast cancers, 95% of renal cell carcinomas, 81% of ovarian cancer, 77% of primary cutaneous melanomas, and 50% of primary uveal melanomas (4, 5, 8, 12). Given their unique overexpression pattern in tumors, NKG2DLs are promising targets for anticancer therapies (4). The widespread presence of NKG2DLs in human cancer indicates that NKG2D-based CAR-T cells have significant therapeutic potential for a wide range of tumor types and broad oncologic applications. This review focuses on cancer immunotherapy harnessing NKG2DR-NKG2DRL axis, including NKG2D-based CAR-T or NK cell therapy and NKG2D-based antibody therapy.

## 2 NKG2D-based CAR-T/NK therapies

**Figure 2:**
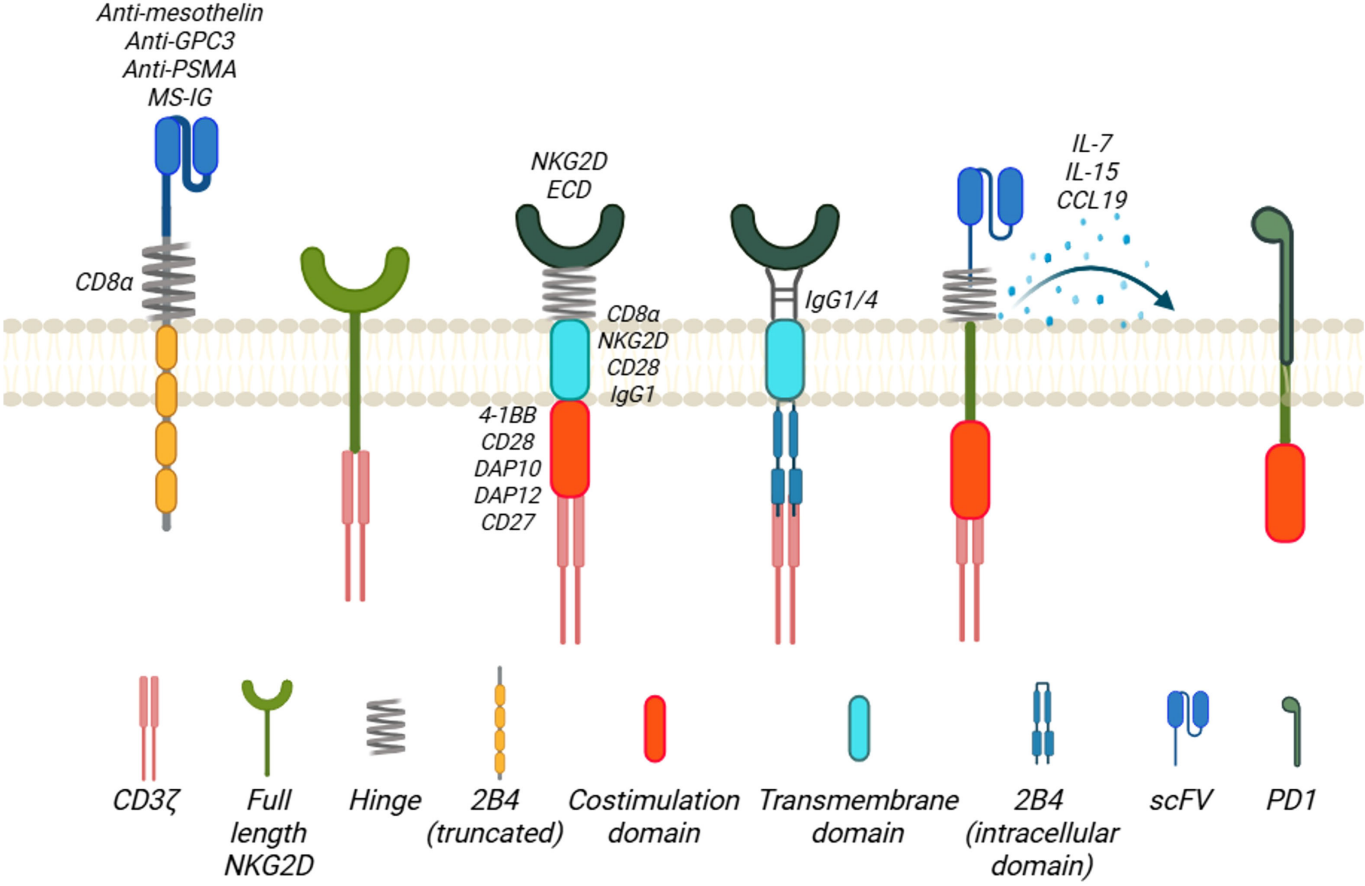
Family of NKG2D-based CAR designs. The antigen-binding domains of the NKG2D-based CARs include extracellular domains of NKG2D receptor molecule (NKG2D ECD) and scFv targeting tumor antigens. The hinge region of the CARs is mostly derived from CD8α molecule, and others are from NKG2D, IgG1/4, or PD1. Except full-length NKG2D or truncated 2B4 is employed in the CAR design, the transmembrane domain of the CARs is CD8α, CD28, or IgG1. For the co-stimulation domains, almost all the NKG2D-based CARs contain CD3ζ, with 4-1BB, DAP12, CD28, DAP10, or CD27 as the second/third co-stimulation domain. Created in BioRender. Chan, W. (2025) https://BioRender.com/777ditl.

Among all cell therapies with NKG2D-based CARs, most of them used T cells rather than NK cells (around 80% of reviewed literature is about CAR-T cells). Although there is no direct comparison on the properties of NKG2D-based CAR-T versus CAR-NK, we can extrapolate from existing knowledge on CAR-T and CAR-NK cells. In general, CAR-T cells have a higher manufacturing efficiency, longer half-life and longer clinical track record. On the contrary, CAR-NK, especially allogeneic off-the-shelf product, offers a more timely and less costly option with additional secondary cytotoxic mechanisms against cancer cells. (**Table 1**). Currently, most of the NKG2D-based CAR constructs are primarily in the 2^nd^ generation design which includes a co-stimulatory domain, either 4-1BB or CD28 plus the intracellular signaling region CD3ζ, although some of those are in the 3^rd^ or above generation with more than 2 co-stimulatory domains (13, 14). Different domains of NKG2D receptor molecules have been utilized in the design of NKG2D CARs and some of the designs are beyond 2^nd^ generation (**Figure 2**). Full-length (1–216) (15–21) or extracellular domain (Uniprot, amino acid from 73 to 216) (1, 22–52) is used as the target binding domain for engaging the NKG2DL-expressing tumor cells including MICA/B and ULBP1–6 for various tumors. Other CARs use transmembrane (Uniprot, amino acid from 7 to 52) (25, 29, 53, 54) of NKG2D. No cytoplasmic domain (Uniprot, amino acid from 1 to 51) of NKG2D was used as a standalone construct in the NKG2D CARs. We elaborate further in the following sections on the use of different NKG2D domains in the CAR design and their application in both preclinical and clinical settings for hematological malignancies and solid tumors.

**Table 1:** A head-to-head comparison of CAR-T and CAR-NK cell characteristics.

Feature	CAR-T	CAR-NK
Adverse Effect	CRS, ICANS, TLS, GvHD if allogeneic cell product	Low potential (rare CRS cases reported), no GvHD with allogeneic product.
Starting cell source	Autologous or allogeneic, PBMCs, UCB, iPSC Fresh or frozen	Autologous or allogeneic, PBMCs, UCB, iPSC Fresh or Frozen
Manufacturing method	Viral and non-viral	Viral and non-viral
Transduction efficiency	High	Low except NK-92
Cytolytic mechanism	CAR-restricted	CAR-dependent and intrinsic innate cytotoxicity via NKG2D, NCRs, etc.
Cellular life span	Long	Relatively short
Vein-to-vein time	Relative longer except ultra-fast CAR-T manufacturing approaches are used	Short due to off-the-shelf capability
Off-the-shelf capability	Low unless genetic modified with TCR/microglobulin knockouts, non-αβ T cells such as gamma-delta T cells or iPSC differentiated T cells are used.	High: expanded NK cells and NK-92 cell line
*In vivo* persistence	High	Low unless IL-15 is present
FDA approved product	Tisagenlecleucel (Kymriah), Axicabtagene ciloleucel (Yescarta), Brexucabtagene autoleucel (Tecartus), Lisocabtagene maraleucel (Breyanzi), Idecabtagene vicleucel (Abecma), Ciltacabtagene autoleucel (Carvykti), and Obecabtagene autoleucel (Aucatzyl).	None
Cost	High	Low due to off-the-shelf capability

CRS, Cytokine Release Syndrome; ICANS, Immune Effector Cell-Associated Neurotoxicity Syndrome; GvHD, Graft-versus-Host Disease; PBMCs, Peripheral Blood Mononuclear Cells; UCB, Umbilical Cord Blood; iPSC, Induced Pluripotent Stem Cells; TCR, T cell receptor.

## 3 CAR targeting NKG2DL

### 3.1 Full length NKG2D

NKG2D receptor as a NK cell activation receptor can lead to NK cell induced cytotoxicity and cytokine production. One of the strategies to harness the intrinsic activation mechanism is to use the full length of NKG2D and studies have attempted to fuse the full length of NKG2D directly to CD3ζ intracellular signaling domains (**Figure 2**). This design has been tested in the preclinical model of ovarian (15), glioma (55, 56), and leukemia models (16–19).

### 3.2 Ectodomain

Most of the NKG2D-based CAR designs replace the single chain variable fragment (scFv) domain of the CAR to the ectodomain of NKG2D, which will bind to NKG2DLs. These designs have been studied across various types of cancers and will be discussed below.

### 3.3 Hinge and transmembrane domains

For the hinge region of all these CARs, they mainly use the region from CD8α (23, 25–28, 30, 31, 33, 35, 39–43, 45, 51, 52, 57–62), CD28 (1, 32, 34, 37, 38, 63, 64), IgG1/4 (24, 32, 34, 37, 38, 46), or NKG2D molecules (25, 29, 53, 54, 65, 66). Notably, 2B4 has been used as a hinge region because it, also known as CD244, is a potent stimulatory co-receptor of NK cell activation and is found to synergize with the NKG2D activation (67). Three different CAR intracellular regions CAR1 (CD244), CAR2 (CD244, NKG2D) and CAR3 (CD244, NKG2D, and CD3ζ) were constructed and compared for their respective functions. CAR2 was found to have a stronger tumoricidal ability on CAR-NK92MI cells and was chosen as the design. PD1, a checkpoint protein in regulating T cell functions and exhaustion, has been used as hinge region of the NKG2D CAR (54). For transmembrane domain, most of the CAR designs adopt CD8α. Others include NKG2D, IgG1 and CD28.

### 3.4 Co-stimulatory domains

All the NKG2D CARs contain the ITAM from the CD3ζ and most of them contain 4-1BB as co-stimulatory domain as well. Other co-stimulatory domains being used include CD28 (25, 34, 40, 64, 68), 2B4 (25, 44, 68), CD27 (44, 68), NKG2D cytoplasmic domain (67) and DAP10/DAP12 (24, 25, 37–39, 47, 66). DAP10, as included in the CAR design (9, 21, 63), is a small transmembrane protein (93 amino acid long) with minimal extracellular domains (63, 69). The cytoplasmic domain of DAP10 contains a short amino acid sequence of YINM (10, 69). Upon activation and tyrosine phosphorylation, the motif will allow the binding of phosphatidylinositol (PI3K) and a Grb2-Vav1-son of sevenless (SOS1) complex (9). This YINM motif is similar to that of CD28, a co-receptor for the co-stimulation of T cell activation with TCR. DAP12 is a small 12-kDa transmembrane protein that consists of 113 amino acids and a single ITAM within its 48-amino acid cytoplasmic domain (69). It shares less than 25% homology with the ITAM motifs found in the human CD3ζ chain and FcϵRI-γ chain. When a DAP12-associated receptor is engaged, it triggers the activation of SRC-family kinases, leading to the phosphorylation of paired tyrosine residues in the ITAM of DAP12 (5, 69). This in turn recruits cytoplasmic ZAP70 tyrosine kinases and initiates downstream signaling and cytokine production. In human immune cells, DAP12 does not associate with NKG2D, but it does form noncovalent complexes with other receptors, such as killer immunoglobulin-like receptors (KIRs) in both human T cells and NK cells. CD27 is a Traf-linked tumor necrosis factor receptor family member and functions as a T cell costimulatory molecule (70). CD27 is required for generation and long-term maintenance of T cell immunity. Instead of using 4-1BB, CD27 cytoplasmic domain was used along with CD3ζ (NKG2D-27z) (44). NKG2D CAR-T cells with the addition of CD27 as a co-stimulatory domain have anti-tumor activity against triple negative breast cancer *in vitro* and *in vivo* MDA-MB-231 fLuc xenograft NOD-SCIDIL2γc-/- (NSG) mouse model. The NKG2D-27z T cells show a persistent phenotype and form a long-term memory in the presence of IL-2.

### 3.5 Armored NKG2D CARs

Beyond 2^nd^ generation of CARs, the NKG2D CARs can partner with other cytokine-induced receptors such as ectodomain of IL-4 receptor and IL-15 receptor (IL-15R) transmembrane+ICD, namely 4/15NKG2D-CAR-T cells (42). IL-15R is a pro-inflammatory cytokine that stimulates NK cell proliferation and expansion, while the IL-4 receptor extracellular domain (ECD) responds to IL-4 in the tumor microenvironment, where inhibitory signals from IL-4R are converted into IL-15R activation signals downstream. C-C motif chemokine ligand 19 (CCL19), a chemokine receptor has also been engineered into the NKG2D CAR design for better trafficking of the CAR cells to the target cells (64). 15×19 NKG2D CAR-T cells, which incorporate the secretion of interleukin (IL)-15 and CCL19, have an augmented cell expansion, promotion of central memory T (T_CM_) cell production, and increased cytotoxicity against gastric cell lines compared to conventional CAR T cells. These CAR-T cells also have reduced expression of T cell exhaustion markers, providing longer cancer surveillance in zebra fish model of gastric cancer. IL-21 connected to the NKG2D CAR constructed with NKG2D ECD, 4-1BB and CD3ζ domains augmented the CAR NK-92 cytolytic functions against lung cancer cell lines A549 and H1975 with increased CD107a, IFN-γ and cell proliferation via activation of PI3K/Akt pathway. This armored CARs also reduce the tumor size in a subcutaneous lung cancer mouse model (71).

## 4 Non-NKG2DL targeting CAR using NKG2D receptor components

Besides targeting NKG2DLs, a group of CAR designs does not use NKG2D ectodomains but uses scFv instead such as anti-mesothelin (25, 63, 67), prostate-specific membrane antigen (PSMA) (67), and glypican 3 protein (GPC3) (63), programmed cell death-1 (PD1) (53, 54) and NKG2D receptor or associated components such as NKG2D transmembrane and DAP10 to diversify the tumor targeting to other tumor-associated antigens.

## 5 Targeting NKG2DL on both solid tumors and hematological malignancies

It is noteworthy that most of the published studies on NKG2D CAR-T/NK therapy have been focused on solid tumors, particularly brain tumors, breast cancer, lung cancers, gastrointestinal tracts, reproductive system, and sarcomas. This trend underscores the significant research interest and potential therapeutic applications of NKG2D CAR-T/NK due to the fact that NKG2DLs are broadly and highly expressed on these solid tumors (12). We summarize and discuss the preclinical studies of NKG2D-based CAR-T or NK cells categorized by cancer types (**Table 2**).

**Table 2:** Preclinical data of NKG2D CAR-T with animal data.

CAR cell type	Disease	CAR construct design	Animal model	Outcome	Reference
CAR-T	Glioblastoma	NKG2D extracellular domain (ECD), CD8 hinge (H) and transmembrane (TM) domain, 4-1BB intracellular domain (ICD), and CD3ζ ICD.	Five to six-week-old B-NDG mice were subcutaneously (s.c.) injected with U-251MG cells (1x10^6^ cells/each) and received CAR-T intravenously (i.v.) (1x10^7^ cells/each) 7 days later	The CAR-T cells greatly reduced U-251MG xenograft tumor burden *in vivo* and do not show significant treatment-related toxicity in the treated mice	(48)
CAR-T	Ovarian cancer	murine CD3ζ ICD and murine NKG2D full length sequence	female B6 mice were intraperitoneally (i.p.) injected with ID8 cells (2- 5x10^6^) and received CAR-T cells (5x10^6^) i.p. 1, 2, and 3 weeks after tumor injection	NKG2D CAR-T cells were able to lead to long-term, tumor-free survival in mice bearing established ovarian tumors. Tumor-free mice were able to reject a rechallenge with ovarian tumor cells 225 days after original tumor injection	(22)
CAR-T	Hepatocellular carcinoma	NKG2D ECD, CD8 hinge and TM, 4-1BB ICD, and CD3ζ ICD.	Five-to-six B-NDG mice were s.c. injected with SMMC-7721 cells (1x10^6^ cells) and received CAR-T i.v. (1x10^7^ cells) 7 days later	NKG2D-BBz CAR-T cells suppressed the growth of SMMC-7721 xenografts	(27)
CAR-T	lymphoma	murine CD3ζ ICD and murine NKG2D full length sequence	C57BL/6 mice were i.v. injected with RMA/RG cells (2- 5x10^6^) and received CAR-T i.v. (7.5x10^6^) days 2, 6, and 10 after tumor injection	CAR-T reduced tumor burdens in both spleens and lymph nodes and prolonged the survival of tumor bearing mice. Multiple treatments with chNKG2D T cells resulted in long-term tumor-free survival. Moreover, these long-term survivors were resistant to rechallenge with wild-type RMA tumor cells (NKG2D ligand–negative)	(19)
CAR-T	Cervical cancer	NKG2D ECD, CD8 hinge and TM, 4-1BB ICD, and CD3ζ ICD.	NOG mice were subcutaneously injected with Siha cells into right flank (4x10^6^) and received CAR-T cells i.v. after 14 days (2x10^6^)	The NKG2D CAR-T could suppress the growth of transplanted tumor and prolong life survival	(30)
CAR-T	glioblastoma	murine CD3ζ ICD and murine NKG2D full length sequence	VM/Dk mice of 6 to 12 weeks were stereotactically implanted with GL-261 cells into the right striatum (2x10^4^) and received CAR-T cells intravenously (5x10^6^) at days 5, 7, and 10 after tumor implantation, or intratumorally (i.t. 2x10^6^) at day 5	NKG2D-based CAR T-cell treatment (i.v. or i.t.) confers a survival benefit in syngeneic orthotopic glioma-bearing mice. Surviving mice were protected long-term against tumor rechallenge. CAR-T cells migrated to the tumor site in the brain after systemic administration	(56)
CAR-T	Breast cancer	NKG2D ECD-CD8 hinge-CD8 TM- CD3ζ(NKG2D-z T. NKG2D ECD-CD8 hinge-CD8 TM-41BB- CD3ζ(NKG2D-BBz. NKG2D ECD-CD8 hinge-CD8 TM-CD27-CD3ζ(NKG2D-27z)	Six-10-week-old female NSG mice were inoculated s.c. on the flank with MDA-MB-231 cells (3x10^6^) on day 0 and received CAR-T later. No information on injection schedule and number of cells	Tumor growth was modestly delayed in mice receiving NKG2D-z T cells. Mice receiving GFP-NKG2D-BBz or NKG2D-27z CAR T cells were protected from rapid progression, which was significantly better than NKG2D-z T cells	(44)
CAR-T	Prostate Cancer	NKG2D ECD-CD8 Hinge-CD8 TM-41BB- CD3ζ (NKG2D-CAR T) NKG2D-CD8 Hinge-CD8 TM-41BB- CD3ζ-T2A-IL7 (NKG2DIL7-CAR T)	Female 6-to-8-week NSG mice were injected s.c. with PC-3 tumor cell (2x10^6^), and received CAR-T i.v. (1x10^7^) when the tumor burden reached approximately 150–200 mm^3^	NKG2D-CAR T cells produced remarkable antitumor ability *in vivo*. Tumor volume and weight were lower in the group treated with NKG2DIL7-CAR T cells than that with NKG2D-CAR T cells	(23)
CAR-T	AML and T-ALL	NKG2D ECD, CD8α hinge, 4-1BB ICD and CD3ζ ICD.	NSG mice were engrafted with 1x10^6^ of Jurkat cells by i.v. injection, then received i.v. infusions of NKG2D-CAR T cells (1x10^7^) at day 3, followed by weekly infusions of 1x10^7^cells/mouse for three weeks, then followed by weekly infusions of 2x10^7^cells/mouse for three additional weeks (6 infusions in total)	NKG2D-CAR T cells reduce tumor progression in a murine model of T-ALL and extend the survival of mice after single or multiple infusions	(72)
CAR-T	Osteosarcoma	NKG2D ECD-CD8 hinge-4-1BB ICD-CD3ζ ICD	Ten- to 12-week-old NSG mice were engrafted with 531MII cells (5x10^5^) by injection through the tibial plateau in the primary spongiosa of both tibias, and received NKG2D-CAR transduced CD45RA-cells(5x10^6^) i.v.	the mice receiving NKG2D CAR-T CD45RA- cells showed lower tumor burden, extended survival and resisted rechallenge	(43)
CAR-T	Liver tumor	NKG2D ECD-CD8 hinge CD8 TM-41BB ICD-CD3ζ ICD-2A-CX3CR1-2A-EGFRt	NSG mice were s.c. injected with 10^5^ HepG2 or A549 cells and received CAR-T cells (3x10^6^) i.v. after 3 weeks, and another infusion of CAR-T 2 months apart	NKG2D-CAR-T co-expressing CX3CR1 reduces tumor burden and extends survival in a liver cancer model, compared with control T cells or IL-15-overexpressing NKG2D CAR-Ts.	(50)
CAR-T	Lung cancer	CD3ζ (ICD)-41BB (ICD)-NKG2D (full length)	Five-to-6-week-old NSG mice were i.p. injected with 5x10^6^ A549 cells, and received 8x10^6^ NKG2D CAR-T cells when the mean fluorescence intensity (MFI) value was >10^9^	NKG2D(bbz) CAR-T cells co-cultured with A549 cells had lower exhaustion and could effectively inhibit tumor growth *in vivo*	(21)
CAR-T	Colorectal cancer	CD8α leader, NKG2D ECD, CD8α hinge, CD28 TM, CD28 ICD, 4-1BB ICD and CD3ζ ICD	NOD/SCID mice were inoculated s.c. with 1x10^6^ HCT-116 cells on the right flank. On day 0 and 7, tumor-bearing mice were treated by the tail vein injection with 1x10^7^ NKG2D CAR-T cells	NKG2D CAR-T reduced tumor volume and extended the survival	(57)
CAR-T	Gastric cancer	NKG2D ECD, CD8α hinge, CD28 TM, CD28 ICD, 4-1BB ICD and CD3ζ ICD. PGK promoter along with IL-15 and CCL19 were introduced into NKG2D-based CARs	HCG-27 cells were stained with a red-fluorescent lipophilic membrane dye and injected into zebrafish 48 hr post-fertilization at a density of 200 cells per fish. CAR-T cells were injected 24 hr postinjection with the same number of HCG-27 cells at the same site	CAR T cells mediate cancer cells oncolysis in zebrafish	(64)
CAR-T	glioblastoma	murine CD3ζ chain cytoplasmic region coding sequence to murine NKG2D full length gene	Mice of 6 to 12 weeks received stereotactic implantation of 2x 10^4^ GL-261, 75x10^3^ CT-2A cells, and mouse mRNA CAR-T cells later. Mouse T cells were electroporated with mRNA encoding either the mNKG2D CAR (CAR) or mIL12 and mIFNa2 (Cyt) or all three mRNAs (CAR+Cyt) using 2.5 mg mRNA for the NKG2D CAR and 0.5 mg mRNA for each cytokine per million cells. Number of CAR-T cells injection is unknown	Multifunctional NKG2D CAR T cells co-expressing mIL12 and mIFNa2 have antitumor activity and confer survival benefit in immunocompetent orthotopic glioma mouse models upon intravenous and local administration	(55)
CAR-T	Pancreatic cancer	CD8α signal peptide, NKG2D ECD, CD8α hinge and TM, 4-1BB (ICD), CD3ζ(ICD), and the shRNA targeting protein 4.1R were subcloned into upstream of NKG2D-CAR	Eight-week-old female NSG mice were inoculated s.c. 6x10^6^ PANC28 cells, and received 1x10^7^ CAR T cells i.v.	4.1R-silencing NKG2D-CAR T cells showed more effective and persistent antitumor activity in mice	(33)
CAR-T	Pancreatic cancer	NKG2D-ECD, CD8 hinge-TM, 41BB(ICD)-CD3ζ(ICD)-T2A-IL-4R ECD-IL15R TM ICD	Eight-week-old NSG mice were injected s.c. with 5x 10^6^ Panc-1 cells, and received CAR-T cells i.v. when tumor size reached 150–200 mm^3^	IL-4/IL-15 NKG2D-CAR-T cells increased the survival of xenograft mice by providing better tumor control	(42)
CAR-T	Colorectal cancer and ovarian cancer	NKG2D-ECD, IgG4 hinge-CD28 TM, 41BB(ICD)-DAP12(ICD)	NSG mice received i.p. injection of 2x10^6^ HCT116 (or 1x10^7^ SKOV3) cells followed by i.p. injection of 1x10^7^ CAR-T on day 7 and day 30	all the mice treated with the NKG2D-DAP12 CAR-T cells survived longer than NKG2D-CD3ζ CAR-T. The incorporation of the DAP12 activation domain may provide a potential clinical advantage in mitigating CRS risk	(37)
CAR-T	Aging	NKG2D ECD, CD8 hinge and TM, 4-1BB ICD, and CD3ζ ICD.	Irradiated or naturally aged (24 months old) C57BL/6 mice were treated i.v. with 1x10^6^ mNKG2D-CAR T. Three rhesus macaques and two cynomolgus macaques (19–22-year-old were i.v. treated with autologous CAR-T cells (1x10^6^ cells/kg)	Mouse NKG2D-CAR T cells alleviated multiple aging-associated pathologies and improved physical performance in both irradiated and aged mice. Autologous T cells armed with the human NKG2D CAR effectively eliminated naturally occurring senescent cells in aged nonhuman primates without any observed adverse effects	(73)
CAR-NK	AML	NKG2D ECD-IgG4 hinge-CD28 TM-41BB ICD-CD3ζ ICD-IRES-IL15	Eight-week NSG mouse were injected i.v. with KG1-Luc cells (5x10^6^), and received NKG2D CAR-NK cells or NKG2D CAR/IL15-NK cells(1x10^7^) i.v. on days 3, 10, and 17	NKG2D CAR/IL15-NK with 3 i.v. injections (days 3, 10, and 17) or 2 injections (days 3, 10) inhibited *in vivo* tumor growth and conferred survival benefit in mouse model	(74)
CAR-NK	Lung cancer	human NKG2D ECD, DAP10 ICD and CD3ζ ICD	Six-week-old male NSG mice were inoculated with 2x10^5^ A549 cells s.c., and receive 5 × 10^6^ NKG2D.CAR-NK-92 cell therapy i.p., once a week, 3 times in total	NKG2D.CAR-NK-92 cells mediated potent anti-tumor responses and significantly reduced the tumor growth rate compared to the PBS group	(75)
CAR-NK	Multiple myeloma	NKG2D ECD-4-1BB ICD-CD3ζ ICD	Eight–to-10-week-old NSG male were i.v. injected with 5 × 10^6^ U-266 cells, and three days later, infused with one single injection of 15 × 10^6^ of CAR-NK cells	*In vivo*, CAR-NK cells mediated highly efficient abrogation of MM growth, and 25% of the treated mice remained disease free	(61)
CAR-NK	neuroblastoma	CD3ζ(ICD)-NKG2D	Twelve- to 16-week-old female NSG mice were implanted s.c. with 1x 10^6^ LAN-1 neuroblastoma cells admixed with 3x10^5^ MDSCs, Ten to 14 days later, when tumors measured at least 100mm^3^, mice were injected i.v. with 5x 10^6^ GD2.CAR-T cells, and 1x10^7^ CAR–NK cells	CAR-NK cells eliminate intratumorally MDSCs which express NKG2D ligands, increased antitumor activity of GD2 CAR-T cells and reduced tumor burden	(76)
Vg9Vd2 NKG2D RNA CAR	Colon cancer Ovarian Cancer	NKG2D-ECD CD8 hinge-CD8TM-CD3ζ	Six to 8 weeks old female NSG mice were i.p. injected with 1x10^7^ HCT116 or SKOV3 cells. On day 7 mice were i.p. injected with 1x10^7^ Vg9Vd2 T cells electroporated with NKG2Dz RNA CAR twice a week for 3 weeks	Vg9Vd2 NKG2D RNA CAR improved the overall survival of tumor bearing mice	(77)
Bispecific antibody, CAR-NK	glioblastoma	NKG2D ECD, CD8α hinge, followed by CD3 TM and ICD.	Female C57BL/6 N 6–8 weeks old were s.c. inoculated with 1×10^6^ GL261/ErbB2 cells, seven days later, were treated by peritumoral injection of 1×10^7^ NKAR-NK-92, with 5μg of NKAB-ErbB2 bispecific antibody. Treatment was repeated two times per week for 3 weeks	NKAR-NK-92 cells and NKAB-ErbB2 bispecific antibody were effective against syngeneic glioblastoma in immunocompetent C57BL/6 mice with prolonged survival	(49)

## 6 NKG2D CAR-T cell targeting solid tumors

### 6.1 Anti-glioma and neuroblastoma

Traditional therapies including chemotherapy, radiation, and surgical resection fail to cure most glioblastoma patients and the median overall survival of GBM patients is only 14.6 months, highlighting an urgent need for new therapeutic options (78). NKG2DLs are highly expressed in GBM and are considered promising targets for CAR-T cell therapy. Dong et al. confirmed that NKG2DLs are highly expressed in human glioblastoma cells, cancer stem cells and tumor samples (3). Besides, the NKG2D-BBz CAR-T cells efficiently kill glioblastoma cells and glioma stem cells *in vitro* and produce high levels of TNF-α, perforin, and granzyme B. The CAR-T cells greatly reduce xenograft tumor burden *in vivo* and do not show significant treatment-related toxicity in the treated mice. As CAR-T cells can pass through blood brain barrier, this study supports the potential of CAR-T therapy as a promising glioblastoma therapeutic strategy.

Neuroblastoma, an extracranial neuroendocrine tumor in pediatric patients, carries a tumor-specific glycolipid antigen GD2 (79). Besides GD2, neuroblastoma cells exhibit high expression levels of NKG2DLs including MICA/B and ULBPs1-3 (80). This could suggest neuroblastoma cells may be susceptible to NKG2D-based CAR-T or CAR-NK cells. Despite this potential, preclinical data evaluating the efficacy of NKG2D-based CAR products against neuroblastoma models remains scarce. Currently, no clinical trials are investigating NKG2D-based CAR-T/NK cell therapies for neuroblastoma treatment.

### 6.2 Anti-liver cancer

A novel NKG2D CAR-T comprising human NKG2D extracellular domain, 4-1BB, and CD3ζ signaling domains (BBz) has been developed to treat hepatocellular carcinoma (HCC) (27). NKG2D CAR-T cells with 4-1BB and CD3ζ efficiently lysed the HCC cell lines SMMC-7721 and MHCC97H *in vitro* in an NKG2DL-dependent manner. The NKG2D CAR-T cells effectively suppressed the growth of SMMC-7721 HCC xenografts. These results indicate that NKG2DBBz CAR-T cells could provide a promising therapeutic option for patients with NKG2DL-positive HCC.

### 6.3 Anti-lung cancers

The introduction of the cytoplasmic domain of DAP10 into second-generation CARs M28z and G28z to create M28z10 and G28z10, targeting mesothelin (MSLN) and glypican 3 (GPC3) respectively, resulted in enhanced and prolonged effector function against MSLN+ lung cancer cell lines (63). In addition, T cells expressing M28z10 or G28z10 exhibited elevated levels of cytokines and show greater anti-tumor activity compared to those expressing M28z. The study demonstrates that DAP10 signaling enhances the function of CAR-T cells in lung cancer cells, indicating its potential to improve the efficacy of CAR-T cell therapies for solid tumors. Jiang J et al. evaluated the therapeutic potential of NKG2D CAR-T cells on non-small cell lung cancer (NSCLC), obtained from diverse human autologous T cell sources (45). The results demonstrated that NKG2D CAR-T cells exhibit significant toxicity with elevated secretion of effector and memory function-related cytokines when compared to non-transduced control T cells. Furthermore, NKG2D CAR-T cells from healthy donors or NSCLC patients’ peripheral blood induced tumor shrinkage, improved survival, increased body weight, increased tumor-infiltrating capacity, and elevated serum IFN-γ levels in mice. This highlights the robust efficacy of NKG2D CAR-T cells in eradicating NSCLC in a NKG2DL-dependent manner, positioning them as a promising therapeutic option for NSCLC patients. An investigation of early cytotoxic lymphocyte infiltration in solid tumors led to the discovery that reduction in C-X3-C Motif Chemokine Ligand 1 & receptor 1 (CX3CL1-CX3CR1) restricts cytotoxic cells from the solid-tumor bed, contributing to tumor evasion (50). To address this, a CAR-T construct was designed, incorporating CX3CR1 overexpression to enhance infiltration. These engineered CAR-T cells demonstrate increased tumor infiltration rates compared to control-activated T cells or IL-15-overexpressing NKG2D CAR-T cells. Furthermore, these CAR-T cells are promising in a liver-cancer model, indicating their potential applicability in various solid malignancies. The combination treatment of NKG2D CAR-NK cells with CD73 targeting demonstrates enhanced anti-tumor cytotoxicity *in vitro* and *in vivo*, indicating a potential alleviation from adenosinergic immune-metabolic suppression (47). Furthermore, the blockade of CD73 improves the intra-tumoral homing of CD56+ CAR-NK cells in human lung cancer xenograft models. This approach represents a pioneering effort to modulate purinergic signaling and enhance adoptive NK cell immunotherapy, shedding light on potential autocrine tumor control and adenosinergic signaling.

### 6.4 Anti-gastrointestinal cancers

Gastric cancer ranks as the fourth leading cause of cancer-related deaths globally and presents a significant challenge in terms of treatment (81). The widespread expression of NKG2D ligands in gastric cancer cells makes them suitable targets for therapy (28). T cells engineered with an NKG2D-based second-generation CAR exhibit significantly enhanced cytolytic activity against gastric cancer cells compared to untransduced T cells. *In vivo*, these engineered cells effectively suppressed the growth of established gastric cancer xenografts either as a standalone therapy or in combination with chemotherapy cisplatin, a chemotherapy drug for treating gastric cancer (28). Another 2nd generation NKG2D-CAR-T cells with Dickkopf WNT Signaling Pathway Inhibitor 1 (DKK1) inhibition on gastric cancers showed the reversal of the suppressive tumor immune environment, increased NKG2DL expression, and significantly enhanced the immune-activating and tumor-killing capabilities of NKG2D-CAR-T cells *in vitro* and *in vivo* (41).

A non-virally engineered NKG2D CAR-T cell showed dose-dependent cytotoxicity against colorectal cancer cells *in vitro*, along with elevated secretion of IL-2 and IFN-γ compared to untransduced T cells (57). In a xenograft model, these cells effectively suppressed tumor growth, reduced tumor sizes, and prolonged overall survival of mice. Importantly, this study demonstrated the infiltration of human NKG2D-positive lymphocytes in tumor sections of treated mice, without severe pathological changes in vital organs, highlighting the safety and potential of NKG2D CAR-T cells as an immunotherapeutic strategy for human colorectal cancer. An mRNA based transient NKG2D CAR-NK cells without DAP10 but CD27/28, enhance NK cell tumor responses significantly against various solid tumor cell lines *in vitro* and demonstrating therapeutic benefits in mice with established colorectal cancer (68). Furthermore, in a clinical trial involving patients with metastatic colorectal cancer, local infusion of the CAR-NK cells resulted in reduced ascites generation, a marked decrease in tumor cell numbers, and rapid tumor regression in the liver region, highlighting the promising therapeutic potential of mRNA based NKG2D CAR-modified NK cells in treating metastatic colorectal cancer (39). Lenalidomide, a 4-amino-glutamyl analogue of thalidomide used as an immunomodulatory drug with potent clinical anti-neoplastic efficacy in solid tumors, significantly increases cytotoxic activity of a second-generation NKG2D-CAR-T cells against colorectal cancer cell lines, HCT116 and SW480 (40).

### 6.5 Anti-pancreatic cancer

Several NKG2D-based CAR-T cells have been investigated in preclinical models of pancreatic carcinoma (PC), which remains a clinical challenge. Cytoskeletal protein 4.1R (4.1R) dampens T cell signaling through inhibiting the phosphorylation of ZAP-70 (82). Knocking down 4.1R activates ERK signaling pathway in NKG2D CAR-T cells, which in turn induces higher cytotoxicity against PC cells *in vitro* and in a xenograft model (33). The NKG2D CAR-T cells with 4.1R knocked down have an increased proliferation rate and reduced expression of inhibitory receptors PD-1 and T-cell immunoglobulin and mucin domain 3 (TIM-3). With the effort to delineate the role of the G-Protein Coupled Receptor 116 (GPR116) receptor on NK cell function and on enhancing antitumor activity, GPR116-/- mice efficiently eliminated pancreatic cancer by enhancing NK cell proportion and targeting PC tumor through the Gαq/HIF1α/NF-κB signaling pathway (83). Furthermore, the study demonstrated that the downregulation of the GPR116 receptor in NKG2D-CAR-NK92 cells enhances their antitumor activity, presenting a novel approach to improve the efficiency of CAR-NK cell therapy. In an orthotopic implantation model for syngeneic pancreatic ductal adenocarcinoma (PDAC) tissue slices which maintains the immunosuppressive microenvironment, NKG2D CAR (chimeric NKG2D full length and CD3ζ)-T cells successfully eliminate myeloid derived suppressor cells (MDSC) and enhance the antitumor activity of subsequently infused CAR-T cells against primary PDAC cells (29). This emphasizes a potential rescue strategy against mechanisms which may impair NKG2D CAR-T cell activity in tumor microenvironment by eliminating MDSCs.

The effectiveness of CAR-T cell therapy is impeded by intrinsic factors within the tumor microenvironment. To address the immunosuppressive signals mediated by interleukin (IL)-4, a novel inverted cytokine receptor (ICR) was designed to convert IL-4R inhibitory signals into IL-15R activation signals downstream (42). This innovative CAR construct, 4/15NKG2D-CAR, co-expresses IL-4R as an extracellular domain and IL-15R as a transmembrane and intracellular domain. This approach augments NKG2D-CAR-T cell efficacy within the pancreatic tumor microenvironment, enhancing their activation, degranulation, cytokine production, and cytotoxicity against IL-4-expressing pancreatic cancer cells. Notably, 4/15NKG2D-CAR-T cells have higher activation, degranulation, cytokine release, and cytotoxic ability against IL-4+ pancreatic cell lines. They also display increased expansion, reduced exhaustion, and a higher proportion of less differentiated T cell phenotypes *in vitro* compared to conventional NKG2D-CAR-T cells. This novel NKG2D-CAR-T cell approach effectively overcomes IL-4-mediated immunosuppression in solid tumors, demonstrating superior tumor eradication compared to conventional NKG2D-CAR-T cells.

### 6.6 Anti-gynecological cancers

Ovarian cancer, an aggressive gynecologic malignancy disease, is ranked the fifth most common cause of women’s cancer deaths in North American, Australian, and Western European populations (84). Novel therapies are needed to either complement or even replace chemotherapy and irradiation. Various NKG2DLs, such as MICA/B and ULBP-1, -2, -3, and -4, are expressed across established ovarian cancer cell lines and primary ovarian cancer samples (30). A study profiling high-grade serous ovarian cancer revealed uniform expression of NKG2DLs on tumor cells, suggesting potential for NK cell-based therapies (85). However, the immunosuppressive tumor microenvironment (TME) is dominated by tumor-associated macrophages (TAMs), MDSCs, and regulatory T cells (T_REG_) (85, 86). These immunosuppressive cells negatively impact the anti-tumor immunity including therapeutic CAR-T or CAR-NK cells via direct cell contact or cytokines such as IL-10 or TGF-β (86). Barber et al. showed adoptive transfer of syngeneic mouse T cells with a CAR which contains only the NKG2D ectodomain and CD3ζ domain, leads to long-term tumor-free survival in ID8 ovarian tumor-bearing mice, generation of both CD4+ and CD8+ ID8-specific T cells, and protection against ID8 tumor challenge and rechallenges (15). These chNKG2D T cells transform the role of myeloid cells within the tumor site and convert them from being immunosuppressive to immune-stimulatory and hence enhance T cell responses. Following chNKG2D T cell treatment, cells isolated from the tumor exhibit increased production of IFN-γ, NO, and other proinflammatory cytokines. The complete response of chNKG2D T cells at the tumor site is dependent on perforin, IFN-γ, and GM-CSF. Spear P et al., reported that a delayed NKG2D CAR-T cell expansion occurs during manufacturing due to the fratricide of NKG2DL expression on activated T cells, but the NKG2D CAR-T eventually expands (26). CD4+ and CD8+ NKG2D CAR-T cells specifically recognize and kill NKG2DL-expressing ovarian cancer cell lines, but not NKG2DL-negative cells. Importantly, this study demonstrates that ovarian cancer cells, which exhibit moderate to low expression levels of NKG2DLs, can be pharmacologically modulated to enhance ligand expression. By using epigenetic agent such as histone deacetylase inhibitor, i.e., sodium valproate (VPA), it can upregulate NKG2DL surface expression and enhance immune recognition by the NKG2D CAR-T cells (31). Administration of these CAR-T cells augments antigen presentation to host CD4+ T cells at tumor site in a CXCR3-dependent manner. These host CD4+ T cells are found to be adequate for optimal tumor protection mediated by NKG2D CAR-expressing T cells but are not necessary if CD4+ T cells are adoptively co-transferred. Besides, there is no obvious off-target toxicity after NKG2D CAR-T infusion. Therefore, NKG2D CAR-T cells become a novel therapeutic approach for treating cervical cancer (30). Target-stimulated secretion of IL-7 from NKG2DIL7-CAR T cells demonstrated increased CAR-T cell number and viability compared to conventional NKG2D-CAR T cells by day 7 due to elevated expression of B-cell lymphoma-2 (Bcl-2), an anti-apoptotic protein, and glucose transporter 1 (Glut1) in NKG2DIL7-CAR T cells (23). This suggests that NKG2D-CAR-T cells expressing IL-7 could potentially persist in the immunosuppressive microenvironment of prostate cancer tissues and induce potent antitumor immunity. A systematic testing on a panel of NK CAR constructs identifies the one with NKG2D transmembrane domain, the 2B4 co-stimulatory domain, and the CD3ζ signaling domain with effective and strong antigen-specific NK cell signaling. Human iPSC-derived NK cells expressing this CAR (NK-CAR-iPSC-NK cells) display a typical NK cell phenotype and demonstrate enhanced anti-tumor activity compared to T-CAR-expressing iPSC-derived NK cells and non-CAR-expressing cells (25). In an ovarian cancer xenograft model, NK-CAR-iPSC-NK cells significantly inhibited tumor growth and prolonged survival, providing a promising “off-the-shelf” standardized lymphocyte therapy for anti-cancer immunotherapy. Similarly, CAR2 (CD244, NKG2D) is chosen to confer a stronger tumoricidal ability on CAR-NK92MI cells (67). p-PSMA-CAR-NK92MI cells are generated by expressing a CAR construct with a polypeptide-based antigen-binding region, an intracellular CD244, and a NKG2D costimulatory domain. They kill PSMA+ target cells selectively and successfully. Additionally, p-PSMA-CAR-NK92MI cells have significantly higher concentrations of IFN-γ, TNF-α, and granzyme B than NK92MI cells. In a CRPC cancer xenograft model, p-PSMA-CAR-NK92MI cells significantly inhibited tumor growth and exerted a more consistent killing effect than NK92MI cells. Ferroptosis is found to be a potential mechanism through which CAR-NK92MI cells utilize to attack cancer cells, which is triggered by IFN-γ.

## 7 NKG2D CAR-T cell targeting hematological malignancies

NKG2D-based CAR-T or CAR-NK cells have been investigated for the treatment of multiple hematological malignancies including mainly acute myeloid leukemia (AML)/myelodysplastic syndrome (MDS) (16–18, 60, 62, 74), T-acute lymphoblastic leukemia (ALL) (17), lymphoma including B (35) or T cell lymphoma (19), and multiple myeloma (MM) (16, 19, 61). An artificial engineered CAR-T cells with an inert NKG2D receptor that can only be activated by specially designed “MicAbodies”, which is an antibody-ULBP2 fusion proteins binding to specific tumor antigens (35). By changing the MicAbody rather than re-engineering the T cells, this CAR-T platform enables the same immune cells to be redirected against different cancer targets as needed. With Burkitt’s lymphoma Raji cells implanted as a solid tumor model, the CAR-T cells could control the tumor burden with significant tumor shrinkages at the dose of 7x10^6^ and 3.5x10^7^. Moreover, high level of NKG2DL is detected on AML cell lines and primary AML cells (87). CAR-T cell products using either NKG2D full length/CD3ζ or NKG2D ectodomain/CD3ζ effectively kill the AML and T-ALL cell lines and primary patient cells (17). The cytotoxicity of NKG2D CAR-T cells is further enhanced by HDAC inhibitor treatment due to the induction of NKG2D-ligand expression in low-expressing AML cells and primary blasts. NKG2D CAR-NK cells generated from primary human NK cells successfully eradicated AML cells both *in vitro* and in a preclinical KG-1 cell-line derived mouse model, with their persistence enhanced by IL-15 co-expression (32). Robust engineering CAR-NK cells using optimized virus-free (88), feeder-free protocols (89) improves the feasibility of translating the CAR-NK cells in clinic (89, 90). A phase I clinical trial on the use of NKG2D CAR-T cells in AML/MDS and MM demonstrated that NKG2D CAR-T cells are safe, with no dose-limiting toxicities, cytokine release syndrome, CAR-T cell-related neurotoxicity, or autoimmune reactions (16). However, clinical benefit was modest, with only temporary responses in AML patients at the highest dose, likely due to limited CAR-T cell persistence and low target density. In a multicenter trial THINK, 16 of 25 AML, MDS, or MM patients were treated with CYAD-01 CAR-T cells at dose-escalation regime (18). At a median follow-up of 118 days, among the 12 evaluable patients with AML or MDS, three (25%) achieved an objective response. Two responding patients with AML subsequently underwent allogeneic hematopoietic stem-cell transplantation after CYAD-01 treatment, achieving durable ongoing remissions of 5 and 61 months. 7 patients (44%) had grade 3 or 4 treatment-related adverse events including cytokine release syndrome in 5 patients, with 1 dose-limiting toxicity reported at dose level three, though no treatment-related deaths occurred. While some anti-leukemic activities are shown in clinical trials, further investigation will focus on the combination strategies to enhance target expression and CAR-T cell persistence.

## 8 Multi-specific NKG2D CAR-T

NKG2D-based CAR-T or CAR-NK cells have been designed to recognize other oncotargets besides NKG2DLs. For AML, dual targeting CAR-T cells have been reported. CD123NK CAR-T cells targeting both CD123 and NKG2DLs shows effective anti-leukemia activity against AML cell lines and in a systemic cell-line derived AML mouse model (60). These NKG2D CAR-T cells also exhibit specific cytotoxicity against primary blasts, myeloid-derived suppressor cells (M-MDSCs), and alternatively activated macrophages (M2 cells), all of which express CD123 or NKG2DLs on over 50% of their cell surface. A novel NKG2D ectodomain/4-1BB/CD3ζ CAR design co-expressing anti-FLT3/4-1BB/CD3ζ CAR has been proposed to target the relapsed/refractory AML patients with FMS-like tyrosine kinase 3-internal tandem duplication (FLT3-ITD), who have limited treatment options and poor prognosis (62). Preclinical studies demonstrated that NKG2D CAR-T cells achieve specific lysis of AML cells both *in vitro* and in a MOLM-13 derived AML xenograft mouse model. Importantly, the efficacy was further enhanced when combined with the FLT3 inhibitor gilteritinib, which upregulates NKG2D ligand expression on AML cells via NFκB2/Rel B signaling pathway. Celyad have developed CD19/NKG2DL, BCMA/NKG2DL and PSMA/NKG2DL multi-specific CAR T-cells to overcome antigen escape and improve anti-tumor efficacy (18, 91, 92). Their products utilize both tandem constructs that comprise the human NKG2D extracellular domain fused to a scFv targeting CD19, BCMA or PSMA, or dual constructs where NKG2D CAR co-expresses anti-CD19, anti-BCMA or anti-PSMA CAR. Celyad showed CD19/NKG2DL multi-specific CAR T-cells are effective *in vitro* against CD19+ and CD19− cell lines and against CD19+ primary B-ALL cells. CD19/NKG2DL tandem CAR T-cells outperform CD19 single CAR-T cells in efficiently controlling tumor cells in a relapsed B-ALL *in vivo* model (93). Similarly, the same study showed BCMA/NKG2DL and PSMA/NKG2DL multi-specific CAR-T cells are efficient even in the absence of BCMA or PSMA. Kaedi Biotherapeutics designs a novel bispecific tandem CAR-T cells (KD-496), which targets both NKG2D ligands and Claudin 18.2 (CLDN18.2) to treat gastric cancer *in vitro* and *in vivo* (94). The bispecific CAR-T cell KD-496 has a CD8 hinge region and transmembrane region, 4-1BB costimulatory region and CD3ζ region. Co-incubation of KD-496 CAR-T cells with double positive NUGC4 and MKN-28-18.2 cells specifically lyse tumor cells even at low effector-to-target (E:T) ratio with elevated IFN-γ secretion. Besides, KD-496 CAR- T cells efficiently eliminate xenograft tumors *in vivo* than single CAR with no obvious safety issue in the treated mice. No obvious pathological changes are observed in the tested organs. Future clinical development of KD-496 CAR-T is warranted with gastric cancer patients. A 2nd generation NKG2D CAR system has been developed recently to have two independent chimeric receptors: One receptor consists of the NKG2D extracellular domain linked with DAP12 for T cell activation, while the other uses the PD-1 extracellular domain linked with 4-1BB for costimulatory signal 2 input (24). The dual NKG2D PDL1 CAR-T cells, generated through electroporation of non-viral piggyBac transposon plasmids, effectively eliminate target cancer cells and eradicate established peritoneal metastasis of both colorectal cancer and ovarian cancer using *in vivo* mouse model.

## 9 Clinical application of NKG2D CAR-T

Based on the information from *clinicaltrial.gov* and open access public domains, there are 21 clinical trials focusing on various NKG2D CAR targets (**Table 3**). These targets encompass a wide spectrum of hematological conditions, including relapsed/refractory AML, MDS, MM, as well as many types of solid tumors such as refractory metastatic colorectal cancer, ovarian cancer, gastric cancer, hepatocellular carcinoma, glioblastoma, medulloblastoma, triple-negative breast cancer, sarcoma, nasopharyngeal carcinoma, and prostate cancer. We summarize those with clinical data reported.

**Table 3:** Clinical data of NKG2D CAR-T.

Trial number	Drug Name	Sponsor name	CAR construct	Indication and stage	Outcome	Reference
NCT02203825	CYAD-01	Celyad Oncology	Autologous CAR T-cell product consisting of full-length human NKG2D receptor fused with the human CD3ξ ICD	AML/MDS and Multiple Myeloma (Phase I)	Four dose levels were evaluated. No DLT, CRS or ICANS was observed	(16)
NCT03415100	NRC-NK-01	Youshan Biomedical Co., Ltd. and The Third Affiliated Hospital of Guangzhou Medical University	Autologous CAR T-cell product consisting of NKG2D extracellular domain and DAP12 ICD	Colorectal cancer (investigator-initiated trial)	Patient, 1001 and, 1002 experienced decrease of ascites generation and reduction of EpCAM-positive cancer cells in ascites samples. Patient, 1003 experienced rapid tumor shrinkage in the liver area observed by ultrasound imaging and PET-CT	(39)
N/A	N/A	La Paz University Hospital	Haploidentical CD45RA depleted cells expressing NKG2D extracellular domain, 41BB and CD3ζ ICD	two pediatric patients with r/r acute leukemia	Although both patients experienced a decrease in peripheral blasts and lactate dehydrogenase after infusions, no clinical benefits were observed	(95)
NCT03018405	CYAD-01	Celyad Oncology	Autologous CAR T-cell product consisting of full-length human NKG2D receptor fused with the human CD3ζ ICD	R/R AML, leukemia, MDS, MM, receiving at least one line of therapy previously	No CRES (CAR T-cell-related encephalopathy syndrome) or deaths related to treatment were reported, and the maximum tolerated dose (MTD) of CYAD-01 was not reached. Three (25%) of 12 evaluable patients with R/R AML or MDS had an objective response	(18)
NCT03370198	CYAD-01	Celyad Oncology	Autologous CAR T-cell product consisting of full-length human NKG2D receptor fused with the human CD3ξ ICD	Unresectable liver metastases from colorectal cancer (LINK)	Terminated	https://clinicaltrials.gov/study/NCT03370198
NCT03692429	CYAD-101	Celyad Oncology	Allogeneic NKG2D CAR-T	Unresectable metastatic colorectal cancer administered after standard chemotherapy	Recruiting	https://clinicaltrials.gov/study/NCT03692429
NCT04991948	CYAD-101	Celyad Oncology	Allogeneic NKG2D-based CAR-T	Metastatic colorectal cancer	Recruiting	https://clinicaltrials.gov/study/NCT04991948
NCT03310008	CYAD-01	Celyad Oncology	Autologous CAR T-cell product consisting of full-length human NKG2D receptor fused with CD3ξ ICD	Colorectal cancer with potentially resectable liver metastases	Unknown status	https://clinicaltrials.gov/study/NCT03310008
NCT05734898		Zhejiang University	NKG2D CAR-NK	R/R AML	Recruiting	https://clinicaltrials.gov/study/NCT05734898
NCT05528341		Xinxiang Medical University	NKG2D-CAR NK92	R/R solid tumor	Recruiting	https://clinicaltrials.gov/study/NCT05528341
NCT06087341		Antonio Pérez Martínez	Memory T cells expressing NKG2D-CAR	Advanced sarcoma	Recruiting	https://clinicaltrials.gov/study/NCT06087341
NCT05213195		Zhejiang University	NKG2D CAR NK	Refractory metastatic colorectal cancer	Recruiting	https://clinicaltrials.gov/study/NCT
NCT05776355		Hangzhou Cheetah Cell Therapeutics Co., Ltd	NKG2D CAR NK	Ovarian Cancer	Recruiting	https://clinicaltrials.gov/study/NCT
NCT04658004		Zhejiang University	NKG2D CAR-T	AML	Not yet recruiting	https://clinicaltrials.gov/study/NCT
NCT05247957		Hangzhou Cheetah Cell Therapeutics	NKG2D CAR-NK	R/R AML	Terminated	https://clinicaltrials.gov/study/NCT
NCT04717999		UWELL Biopharma	NKG2D CAR-T	Recurrent glioblastoma	Unknown status	https://clinicaltrials.gov/study/NCT
NCT04324996		Chongqing Public Health Medical Center	NKG2D-ACE2 CAR-NK secreting super IL15 superagonist and GM-CSF neutralizing scFv	COVID-19	Unknown status	https://clinicaltrials.gov/study/NCT
NCT05248048		The Third Affiliated Hospital of Guangzhou Medical University	NKG2D CAR-T	Previously Treated Liver Metastatic Colorectal Cancer	Unknown status	https://clinicaltrials.gov/study/NCT
NCT06379451		Changzhou No.2 People’s Hospital	NKG2D-CAR-NK	R/R Multiple Myeloma	Not yet recruiting	https://clinicaltrials.gov/study/NCT06379451
NCT04270461		Jiujiang University Affiliated Hospital	NKG2D CAR-T with CD8 hinge region and TM region, 4-1BB ICD and CD3ζ ICD	r/​r NKG2DL+ Solid Tumors	Withdrawn	https://clinicaltrials.gov/study/NCT04270461
NCT06503497		Zhejiang University	Second-line systemic chemotherapy sequential NKG2D CAR-NK cell therapy	Pancreatic cancer	Not yet recruiting	https://clinicaltrials.gov/study/NCT06503497
NCT05131763		Fudan University	NKG2D CAR-T with CD8 hinge region and TM region, 4-1BB ICD and CD3ζ ICD	r/​r NKG2DL+ solid Tumors	Unknown status	https://clinicaltrials.gov/study/NCT05131763
NCT03415100		The Third Affiliated Hospital of Guangzhou Medical University	Autologous or allogeneic NK cells transfected by mRNA electroporation	Metastatic solid tumors	Unknown status	https://clinicaltrials.gov/study/NCT03415100
NCT06478459		Zhejiang University	Intratumoral NKG2D CAR-NK Cell injection guided by endoscopic ultrasound	Advanced pancreatic cancer	Not yet recruiting	https://clinicaltrials.gov/study/NCT06478459
NCT04550663		The Affiliated Nanjing Drum Tower Hospital of Nanjing University Medical School	NKG2D CAR-T	r/​r NKG2DL+ solid tumors	Unknown status	https://clinicaltrials.gov/study/NCT04550663
NCT05382377		Jianming Xu	NKG2D CAR-T	Advanced NKG2DL+ solid tumors	Recruiting	https://clinicaltrials.gov/study/NCT05382377
NCT05583201		Jianming Xu	NKG2D/CLDN18.2-based CAR-T	Advanced NKG2DL+/​CLDN18.2+ solid tumors	Recruiting	https://clinicaltrials.gov/study/NCT05583201
NCT06134960		Peking University	NKG2D/CLDN18.2-based CAR-T	Advanced NKG2DL+/CLDN18.2+ solid tumors	Not yet recruiting	https://clinicaltrials.gov/study/NCT06134960
NCT06509490		Cancer Institute and Hospital, Chinese Academy of Medical Sciences, Beijing, China	NKG2D CAR-T	Advanced NKG2DL+ solid tumors	Recruiting	https://clinicaltrials.gov/study/NCT06509490
NCT06193902		Leucid Bio	Lateral NKG2D CAR-T with complementary signaling domains integrated in parallel across the cell membrane	NKG2DL-expressing solid tumors	Recruiting	https://clinicaltrials.gov/study/NCT06193902
NCT05976906		Zhejiang University	Dual-target NKG2D-NKp44 CAR-T	Advanced solid tumors	Recruiting	https://clinicaltrials.gov/study/NCT05976906
NCT04623944		Nkarta, Inc.	Allogeneic CAR NK with NKG2D activating receptor, OX40 costimulatory domain, CD3ζ ICD, membrane bound IL-15	AML or MDS	Active, not recruiting	https://clinicaltrials.gov/study/NCT04623944
NCT05837299		Changhai Hospital	NKG2D CAR-T	CLDN18.2 positive advanced solid tumors	Recruiting	https://clinicaltrials.gov/study/NCT05837299
NCT04107142		CytoMed Therapeutics	Allogeneic NKG2DL-targeting CAR-γΔ T	R/R solid tumor	Unknown status	https://clinicaltrials.gov/study/NCT04107142

A study team based in in Guangdong, China reported a phase 1 clinical trial results on three metastatic colorectal cancer patients received adoptive transfer of NKG2D CAR mRNA-engineered natural killer cells (39). CAR-NK cells are prepared by electroporation of *in vitro* transcribed mRNA NKG2D CAR with NKG2D ectodomain fused to DAP12 (39). These NKG2D mRNA CAR-NK shows strong cytotoxicity against tumor cells *in vitro* and in mouse models. A patient received two intraperitoneal injections (i.p.) of autologous CAR-NK cells (2x10^7^ and 1x10^8^ cells), while another patient was treated with four i.p. infusions of allogeneic haploidentical CAR-NK cells (1x10^8^, 3x10^8^, 5x10^8^, and 7x10^8^ cells). Both patients had decreased ascitic fluid production with reduced EpCAM-positive cancer cells in ascites samples. The 3^rd^ Patient was treated with six infusions of allogeneic haploidentical CAR-NK cells (5x10^8^ cells twice, 1x10^9^ cells twice, 2x10^9^ cells twice), with ultrasound-guided percutaneous injection, followed by intraperitoneal infusion of the CAR-NK cells. Rapid tumor shrinkage in the hepatic area was demonstrated by ultrasound imaging and positron emission tomography (PET)-computed tomographic (CT), which was confirmed by immunohistochemistry staining.

In a study performed on two pediatric patients with advanced relapsed and refractory acute leukemia, haploidentical CD45RA depleted cells which expressed NKG2D-41BB-CD3ζ CAR were infused (58). Patient 1 was a three-year-old female who received 3 weekly infusions of NKG2D CAR-TCD45RA- (1x10^7^ cells/kg), and the only adverse effect was fever. Then the patient underwent lymphodepletion prior to two weekly infusions of the same number of CAR-T cells, with only skin rash observed. However, the patient died on day +60 post-infusion due to disease progression. Patient 2 was a 15-year-old female, who received a single dose of CAR-T CD45RA- (1x10^7^ cells/kg), experienced grade 2 cytokine release syndrome (CRS), and died on day +14. Patients 2 had an invasive fungal infection, which might impact on the outcome of the patient. Although a decrease in peripheral blasts and lactate dehydrogenase was observed after infusions in both patients, no clinical benefits were observed.

CYAD-01 from Celyad Oncology (formerly known as NKR-2) is an autologous NKG2D-CAR T cell therapy being tested alone or in combination with chemotherapy for hematological and solid cancers. An enhanced version of CYAD-01, CYAD-02, incorporates the shRNA targeting NKG2D ligands on CAR-T cells to improve its efficacy and is currently under clinical trials in patients with acute myeloid leukemia and myelodysplastic syndrome (13, 96). Additionally, Celyad Oncology has developed CYAD-101, an allogeneic NKG2D-CAR-T cell therapy for patients with unresectable metastatic colorectal cancer. A single infusion of low-dose CYAD-01 in the absence of preconditioning chemotherapy was evaluated in a first-in-human clinical study (NCT02203825) (10). Autologous T cells were transfected with a γ-retroviral vector encoding NKG2D CAR with CD3ζ signaling domain. Four dose levels (1×10^6^, 3×10^6^, 1×10^7^, 3×10^7^ total viable T cells) were evaluated. No dose limiting toxicity (DLT), CRS or Immune effector cell-associated neurotoxicity syndrome (ICANS) was observed, none of the grade 3 and 4 adverse events were attributed to the NKG2D-CAR-T infusion. No objective tumor responses were observed in the low dose group. Only one patient with AML at dose level 4 experienced improvement in hematologic parameters without further treatment. The expansion and persistence of NKG2D-CAR T cell was limited according to preclinical study, suggesting higher dose and multiple infusions of CYAD-01 might be necessary (16).

Celyad’s allogeneic CAR-T pipeline, CYAD-101, featured the incorporation of the TCR Inhibitory Molecule (TIM) to mitigate the risk of graft-versus-host disease (GvHD). TIM, a truncated CD3ζ peptide, is co-expressed with the NKG2D-CAR construct and integrated into the T cell receptor (TCR) complex to dampen TCR responses. Notably, CYAD-101 showed CAR-driven antitumor activity both *in vitro* and *in vivo* with signs of GvHD in mouse models (92). A significant aspect of the CYAD-101 pipeline is the production of two clinical grade independent batches from a single donor, resulting in 4.8×10^10^ CAR-T cells, an ample quantity for the entire dose-escalation phase of the planned alloSHRINK clinical trial (NCT03692429). These two batches exhibited high consistency, predominantly comprising a CD4+ T-cell population that maintains a similar effector memory/central memory phenotype with minimal expression of exhaustion markers (over 99% LAG3-PD-1- population). Furthermore, these clinical grade CYAD-101 cells demonstrated specific *in vitro* anti-tumor activity with minimal response to TCR stimulation.

## 10 NKG2D-based antibodies as cancer treatment

The anti-tumor role of NKG2D has been demonstrated in various studies using different anti-NKG2D antibodies either as a neutralizing antibody or activation antibody. Using two novel anti-mouse NKGD monoclonal antibodies (derived from hamster), stimulation with anti-NKG2D mAb redirected and enhanced lysis of tumor targets expressing NKG2D ligand (97). Notably, NKG2D alone did not induce cytokine release, but in conjunction with other NK activation receptors, cytokine release can then be enhanced. This supports NKG2D’s ability to co-stimulate multiple NK activation receptors. Cross-linking NKG2D with an anti-NKG2D antibody to simulate ligand binding shows an increase in the production of soluble TRAIL (sTRAIL) by γδ T cells (98). This sTRAIL induces apoptosis in lung cancer cells through TRAIL R2. Neutralizing these sTRAIL or blocking lung cancer cell TRAIL R2 leads to a significant reduction in γδ T-cell-mediated cytotoxicity to lung cancer cells, suggesting the unresolved mechanism of anticancer immunity through the NKG2D-regulated production of sTRAIL. Talebian L et al. also proved the role of NKG2D by demonstrating that via blocking the NKG2D receptor through monoclonal antibodies or siRNAs on cytotoxic T cells will reverse their cytotoxicity on autologous myeloma cell (99). In the same study, the T cell population of NKG2D+CD3+CD8+ can be expanded *ex vivo*, and these cells identify and destroy autologous and allogeneic myeloma cells independently of T-cell receptor or MHC-I expression. NKG2D+CD3+CD8+ T cells provide anti-myeloma activity in a NKG2D-dependent manner and trigger the release of proinflammatory IFN-γ and TNF-α.

Two bispecific antibodies have attempted to target NKG2D ligand negative tumor cells. NKAB-ErbB2 significantly enhanced the lysis of ErbB2-positive breast carcinoma cells by NKG2D-expressing NK cells from peripheral blood, surpassing the effectiveness of an ErbB2-specific IgG1 mini-antibody that induces cytotoxicity via CD16 activation (49). Additionally, NKAB-ErbB2 demonstrates synergy with NK-92 cells or primary T cells engineered with an NKG2D-CD3ζ chimeric antigen receptor (NKAR), leading to targeted cell killing and notably improved anti-tumor activity. Importantly, these effects are not hindered by soluble MICA, which is known to inhibit NKG2D-mediated natural cytotoxicity. In an immunocompetent mouse model of glioblastoma with low or absent NKG2DL expression, the combination of NKAR-NK-92 cells and NKAB-ErbB2 effectively suppressed the growth of ErbB2-positive tumors, resulting in treatment-induced endogenous antitumor immunity and cures majority of the animals. A novel NKG2D bispecific antibody (CS1-NKG2D biAb) is designed to bridge CS1 (other name SLAMF7) positive human MM cell lines and all NKG2D+ cytolytic cells including NK and cytotoxic T cells (6). The cytotoxicity was specific to both CS1 and NKG2D with a specific triggering on the phosphorylation of AKT, a downstream protein kinase of the activated NKG2D-DAP10 complex on effectors cells. *In vivo*, the survival is significantly extended using the CS1-NKG2D biAb in a xenograft NSG mouse model engrafted with both human PBMCs and MM cell lines.

## 11 Synergism with OTHER CANCER therapy or antibodies

The effect of anti-NKG2D antibody has been tested also in the presence of chemotherapy or other antibodies targeting immune checkpoint protein. Using A549 lung cancer and murine Lewis lung carcinoma models, applying either anti-PD-1 or anti-NKG2D antibodies in combination with oxaliplatin (OXA) synergistically suppresses tumor growth and prolongs mouse survival, offering a promising treatment benefit (100). OXA’s role in promoting T cells and NK cells infiltration through the CXCL9/10/11-CXCR3 axis can enhance anti-PD1 or anti-NKG2D immunotherapy in lung cancer. Ionizing radiation (IR) induced NKG2D ligand RAE-1 expression *in vivo*, and the combination therapy of IR and anti-CTLA-4 mAb resulted in tumor-infiltrating lymphocyte (TIL) motility arrest. However, the addition of anti-NKG2D mAb blocked this TIL arrest induced by the IR/9H10 combination therapy.

## 12 Challenge facing NKG2D-based immunotherapeutics

While early results are promising, potential challenges remain to be resolved. For instance, the presence of surface NKG2DLs does not always translate into an enhanced cytolytic immune response against cancer due to NKG2DLs shedding and tumor microenvironment. Soluble NKG2DLs in the serum of patients with leukemias, MM, and lymphomas have been shown to have prognostic significance (101–104). Tumors shed NKG2DLs from their surface to evade the immune surveillance, resulting in high levels of soluble NKG2DLs (5, 10). These soluble NKG2DLs bind to NKG2D, leading to internalization and systemic desensitization of NKG2D in effector cells and impaired anti-tumor function. Therefore, although targeting NKG2DLs represents a promising treatment strategy for cancer therapy, it is crucial to address the potential impact of soluble NKG2DLs on effector cell responsiveness by downregulating the NKG2D. Strategies including pharmacological modulation have been proposed to mitigate the effect of shedding. Inhibitors of ADAM10 and ADAM17, which function as ectodomain sheddases to cleave the NKG2D from effector membrane, have been proposed to block and restore the NKG2D activation (105, 106). Nutlin-3a, a small molecule inhibitor targeting the p53-antagonist MDM2, reverses the dysregulation of p53 functions in neuroblastoma cell lines and patient cells (107). This treatment stimulates the surface expression of ULBPs and NK cell coreceptor DNAM-1 ligands (PVR and Nectin-2). As a result, Nutlin-3a induces augmented NK cell cytotoxicity against neuroblastoma both *in vitro* and *in vivo*. This evidence also suggests the potential integration of different pathways regulating NKG2DL and DNAM-1 ligand expression. Dual-targeting CAR designs incorporating both NKG2D and DNAM-1 (108) warrant further clinical investigation.

During CAR-T expansion, CD3/CD28 and cytokine-induced activation creates stress and transiently induces NKG2DL on T cells (18). Consequently, NKG2D CAR-T cells may kill each other during culture and expansion, preventing the large-scale manufacturing of CAR-T for clinical development. Potential strategies to avoid fratricide have been proposed in the large-scale manufacturing of NKG2D CAR-T (109). PI-3K inhibitor LY294002 was included in the manufacturing process. LY294002 is shown to reversibly reduce NKG2D expression on the CAR-T surface and partially controls fratricide during manufacturing and enhanced viability post-thawing as well. But the level of manufacturing failure increases, as the trial moved through the dose-escalation phase towards the upper dose level. This is largely attributed to the effect of LY294002 which can suppress T cell proliferation (110). Alternatively, NKG2D blocking antibody was added during the expansion phase of cell culture to further prevent fratricide (109). These two strategies enable the efficient manufacture of CYAD-01 T cells to the levels required for the THINK clinical trial. NKG2D CAR-NK instead of CAR-T cells are reported to be resistant to the soluble NKG2DLs and self-fratricide because NKG2DLs expresses on T cells upon activation and CAR-T manufacturing under cytokine and CD3/CD28 stimulations (76).

TME influences NKG2D-based CAR-T or CAR-NK efficacy through multiple mechanisms such as immunosuppressive cells like TAMs, MDSCs and T_REG_, which express NKG2DLs, enabling CAR-mediated targeting of both malignant and stromal components (13, 14). In a preclinical neuroblastoma model, NKG2Dζ NK cells effectively kill both ex vivo generated MDSCs *in vitro* and tumor-infiltrating MDSCs *in vivo*. This elimination of immunosuppressive MDSCs indirectly reduces tumor burden and prolongs survival (76). However, the TME also poses challenges through soluble NKG2D ligands (e.g., MICA/B) and exosomal factors that downregulate receptor expression or induce exhaustion (13), although NKG2Dζ NK are reported to be resistant to the immunosuppressive TGF-β and soluble NKG2DL in TME of neuroblastoma (76). Metabolic constraints and inhibitory checkpoints (e.g., PD-1, LAG-3) further dampen functionality, though combination strategies with radiation therapy, HDAC inhibitors or checkpoint blockade show potential to enhance ligand expression and sustain effector responses (13, 17, 111, 112). While off-tumor toxicity remains a concern due to ligand expression on stressed non-malignant tissues, current clinical approaches aim to balance efficacy with safety through controlled CAR designs and microenvironment modulation.

NKG2DL, as a stress ligand, expresses in cells under stress response and cell senescence. The on-target-off-tumor effect of NKG2D CAR-T cells on non-malignant cells under stress or senescence during stressful cancer treatment remains unknown. Evidence shows NKG2D CAR-T cells could eliminate human cells undergoing senescence induced by replicative stress, oncogenic stress, DNA damage *in vitro* in a selective and effective way (73).

## 13 Targeting NKG2D using blocking or activating antibodies

A set of highly specific anti-human NKG2D single-domain antibodies targeting different epitopes has been developed over the years (113, 114). These single-domain antibodies are incorporated into bivalent and bispecific antibodies using a versatile plug-and-play Fab-like format. Depending on the context, these Fab-like antibodies display activating or neutralizing effects on the immune response mediated by the NKG2DL/NKG2D axis. In solution, the bivalent anti-NKG2D antibodies, which compete with NKG2DL, effectively block the activation of NK cells seeded on immobilized MICA, making them potential antagonists. Additionally, a bispecific anti-NKG2DxHER2 antibody that simultaneously engages HER2 on tumor cells and NKG2D on NK cells induces cytotoxicity of unstimulated NK cells in a tumor-specific manner, regardless of their apparent affinities and epitopes (49). Crucially, the bispecific antibody that does not compete with ligand binding retains its full cytotoxic activity in the presence of ligands, which is a valuable attribute for overcoming immunosuppressive effects induced by soluble ligands in the tumor microenvironment. Recent studies have highlighted the importance of 2:1 stoichiometry, distinct binding epitopes from natural ligand, and optimal-affinity interactions (nM range) in the design of agonistic anti-NKG2D antibodies (115, 116).

## 14 NKG2D as AN activation receptor or coreceptor depending on effector cell types

Although NKG2D is primarily recognized as an activation receptor in NK cells, it also demonstrates versatility by functioning as a co-receptor depending on the cell type and context. As an activating receptor, NKG2D is predominantly expressed on cytolytic cells in the immune system, where its engagement can directly stimulate the production of cytokines and cytotoxic molecules in NK cells. On activated NK cells primed by pro-inflammatory cytokines such as IL-2 and IL-15, NKG2D provides direct stimulatory signals. However, NKG2D’s role extends beyond just activation. In resting NK cells, it acts as a co-activator, synergizing with other receptors such as NKp46 and 2B4 (117). In αβ T cells such as CD8+ T cells, NKG2D typically provides a co-stimulatory signal, promoting T cell receptor (TCR)-dependent cytotoxicity, production of pro-inflammatory cytokines, and memory differentiation (99). In invariant NKT cells, it facilitates direct target cell lysis and provides co-stimulatory activation, whereas in mucosal-associated invariant T (MAIT) cells, particularly CD8+ subsets, it functions primarily as a co-stimulatory molecule. This dual functionality underscores NKG2D’s status as one of the most versatile and widely distributed activating/co-stimulatory NK-related receptors. The activation signals mounted on effector cells depend on the intensity and duration of ligand engagement, further highlights its adaptability. Ultimately, NKG2D’s role can vary based on the cell type, activation state, and surrounding cytokine environment, making it a crucial and flexible component of the immune system’s regulatory network.

## 15 Conclusions and future directions

NKG2D has been harnessed in numerous CAR and antibody designs for cancer immunotherapy, incorporating full-length receptors, extracellular domains, or cytoplasmic components, and applying these constructs to both T and NK cells. With the known intrinsic functions in NKG2D-NKG2DL axis, further investigations should focus on how these NKG2D-based CARs and antibodies may affect the intrinsic signaling pathways inside the effector cells. Utilizing NKG2D-knockout cellular models in NK cell lines such as NK-92 and KHYG-1, as well as in primary NK cells, represents a promising strategy to investigate the intrinsic effects of NKG2D-CAR expression and antibody engagement on NK cell function. While emerging evidence supports the therapeutic potential of NKG2D-based CARs and antibodies in cancer immunotherapy, their application will be particularly promising for solid tumors, where NKG2D ligands are often highly expressed.
